# Development of Mouth Dissolving Tablets of Clozapine Using Two Different Techniques

**DOI:** 10.4103/0250-474X.44611

**Published:** 2008

**Authors:** R. S. Masareddy, R. V. Kadia, F. V. Manvi

**Affiliations:** Department of Pharmaceutics, K. L. E. S's College of Pharmacy, Nehrunagar, Belgaum-590 010, India

**Keywords:** Clozapine, direct compression, sublimation, mouth dissolving

## Abstract

Mouth dissolving tablets constitute an innovative dosage form that overcomes the problems of swallowing and provides a quick onset of action. In view of enhancing bioavailability an attempt has been made to study two different methods direct compression and sublimation in formulation of mouth dissolving tablets of clozapine. Total four formulations using various superdisintegrants and subliming agents were prepared. All prepared formulations were evaluated for physico-chemical parameters. The formulations exhibited good disintegration properties with total disintegration time in the range of 25 to 35 s. Comparative evaluation of two methods showed direct compression method is a better alternative to sublimation method as its formulations rapidly disintegrate in oral cavity. *In vitro* cumulative percentage drug release for formulations prepared by direct compression with explotab superdisintegrants shows 99.79 while sublimation method using camphor 93.58 release in 12 min. Kinetic studies indicated that all the formulations followed first order release with diffusion mechanism.

The oral route of administration is considered as the most widely accepted route. But the most evident drawback of the commonly used oral dosage forms like tablets and capsules is difficulty in swallowing, leading to patients incompliance particularly in case of pediatric and geriatric patients^1^. Thus, a new delivery system known as oral fast dissolving/disintegrating (FDDS)/melt-in-mouth tablets gaining importance. These oral dosage forms dissolve rapidly in saliva and can be swallowed without the need of drinking water^2^. Elimination of bitterness is an important criterion in product formulation of mouth dissolving tablets^3^.

Superdisintegrants added in the formulation increase the dissolution characteristics thus increasing the bioavailability of drug^4^. Mouth dissolving tablet disintegrate in mouth and are useful for potent drugs where fast absorption is required. Clozapine, an antipsychotic agent has been found to be an ideal candidate for mouth dissolving tablets^5^. Clozapine is used to suppress both positive and negative symptoms of schizophrenia and many neuroleptic responses.

In the present study, an attempt has been made to develop mouth-dissolving tablets of clozapine by direct compression and sublimation methods using suitable superdisintegrants and subliming agents.

Clozapine was procured from Sun Pharmaceuticals Pvt. Ltd. Vadodara, India. Flowcel PH 102 (MCC) was obtained from Gujarat Micro Wax Ltd. Indore, India. Kollidon-CL was obtained from Signet Chemical Corp. Mumbai. Exlpotab was obtained from Forum Bioscience, London. Camphor and ammonium carbonate were obtained from S. D. Fine Chemicals, Mumbai. All other ingredients were of analytical grade.

Clozapine tablets each containing 50 mg drug was prepared as per the formulae given in Table 1. The formulations F1and F2 were prepared by the direct compression method^6^ and the formulations F3 and F4 were prepared by sublimation method^7^. In direct compression method all the ingredients were passed through # 60 sieve. Clozapine, mannitol, microcrystalline cellulose and sodium saccharin were triturated in a glass mortar. Superdisintegrants were incorporated in the powder mixture and finally magnesium stearate and talc were added as lubricant. The powder mix was weighed individually and compressed with 10 mm flat face surface punches using hydraulic press. In sublimation method accurately weighed quantities of clozapine, volatile component, mannitol and sodium saccharine were mixed and passed through # 45 sieves. Finally magnesium stearate and talc were added and then subjected to compression. After compression tablets were heated in hot air oven at 60°until constant weight was obtained to ensure the complete removal of volatile component. The tablets were evaluated for various parameters such as physical appearance, weight variation, hardness, friability, drug content uniformity, disintegration time and *in vitro* dissolution test.

**TABLE 1:** COMPOSITION OF MOUTH DISSOLVING TABLETS OF CLOZAPINE

Ingredients	F1 (mg)	F2 (mg)	F3 (mg)	F4 (mg)
Clozapine	50	50	50	50
Avicel PH-102	100	100	--	--
Mannitol	101	101	179	179
Kollidon-CL	38	--	--	--
Explotab	--	38	--	--
Sodium saccharine	5	5	5	5
Camphor	--	--	60	--
Ammonium bicarbonate	--	--	--	60
Magnesium stearate	3	3	3	3
Talc	3	3	3	3

Formulation F1 and F2 uses direct compression method and formulation F3 and F4 uses sublimation method.

Tablet containing 50 mg of drug was dissolved in 100 ml of 0.1 N hydrochloric acid in volumetric flask. The drug is allowed to dissolve in the solvent. The solution was filtered, 1 ml of filtrate was taken in 50 ml of volumetric flask and diluted up to mark with 0.1N hydrochloric acid and analyzed spectrophotometrically at 209 nm. The concentration of clozapine in mg/ml was obtained by using standard calibration curve of the drug.

The *in vitro* disintegration time was determined using disintegration test apparatus. A tablet was placed in each of the six tubes of the apparatus containing saliva fluid as the immersion liquid; one disk was added to each tube. The time in seconds taken for complete disintegration of the tablets with no palpable mass remaining in the apparatus was measured in seconds.

The method reported by Yunixia *et al.* was used to measure tablet wetting time. A piece of tissue paper folded twice was placed in a small petri dish (6.5 cm) containing 6 ml of simulated saliva pH, a tablet was placed on the paper and the time for complete wetting was measured. Three trials for each batch were performed and standard deviation determined. A tablet was placed in 10 ml of simulated saliva fluid. Time required for complete dispersion of tablet was measured in seconds.

*In vitro* release studies were carried out using USP XXIII tablet dissolution test apparatus paddle method at 37±1^0^, taking 900 ml of saliva fluid as dissolution medium. Speed of rotation of the paddle was set at 50 rpm. Aliquots of 5 ml were withdrawn after 2, 4, 6, 8, 10 and 12 min and analyzed spectrophotometrically at 209 nm.

The present study was carried out to prepare mouth-dissolving tablet of clozapine by two different methods, direct compression using superdisintegrants kollidon and explotab in direct compression and camphor and ammonium bicarbonate as subliming agents in sublimation method (fig. 1). A total number of four formulations were prepared and evaluated. The prepared tablets in all the formulations possessed good mechanical strength with sufficient hardness in the range of 2.38 kg/sq cm to 4.16 kg/ sq cm. Percent friability was found to be less than 1% in all formulations indicating tablets were mechanically stable. Formulations prepared by sublimation method were more friable in comparison with those prepared by direct compression method. All the prepared formulation passed weight variation test, with percent weight variation within the pharmacopoeial limits of ±7.5% of the average weight. The percent drug content of all the tablets were found to be between 99.06 to 99.8% of clozapine. Further, the tablets were subjected for the evaluation of *in vitro* disintegration time. All the formulations showed disintegration time less than 30 s. Formulations prepared by direct compression disintegrated more rapidly in comparison to that formulated using sublimation method. Wetting time corresponds to the time taken for the tablet to disintegrate when kept motionless on the tissue paper in a petri dish. This method will duplicate the *in-vivo* disintegration, as the tablet is kept motionless on the tongue. The value lies between 16.76±1.27 to 25.5±0.73 s. *In vitro* dispersion time taken by the tablet for complete dispersion is measured. The time for all formulations varied between 18.0±1.0 to 35.3±0.86 s. Formulations F1 and F2 prepared by direct compression release 89.89%, 99.79% drug while F3 and F4 prepared by sublimation releases 93.58% and 98.33%, respectively at the end of 12 min. The rapid drug dissolution might be due to easy breakdown of particles and rapid absorption of drugs into the dissolution medium. Formulations prepared by sublimation disintegrated due to porous structure formation after sublimation of camphor and slope values signify that the release rate follows first order kinetics.

**Fig. 1:**
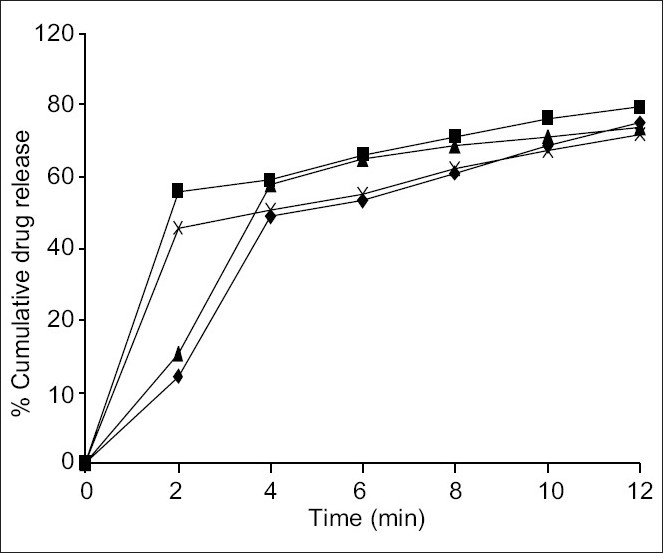
*In vitro* cumulative percent release of clozapine in 0.1N hydrochloric acid. Drug release from formulation F1 (-♦-) containing kollidon-CL, F2 (-■-) containing explotab as superdisintegrants, F3 (- ▲ -) containing camphor and F4 (-x-) containing ammonium bicarbonate as sublimating agents.

From the study, it can be concluded that direct compression method showed better disintegration and drug release as compared to sublimation method. The prepared tablets disintegrate within few seconds without need of water; thereby enhance the absorption leading to its increased bioavailability.
